# How can asset-based approaches reduce inequalities? Exploring processes of change in England and Spain

**DOI:** 10.1093/heapro/daae017

**Published:** 2024-03-02

**Authors:** Viola Cassetti, Katie Powell, Amy Barnes, Tom Sanders

**Affiliations:** Sheffield Centre for Health And Related Research (ScHARR), University of Sheffield, Regent Court, S1 4DA, Sheffield, UK; Sheffield Centre for Health And Related Research (ScHARR), University of Sheffield, Regent Court, S1 4DA, Sheffield, UK; Sheffield Centre for Health And Related Research (ScHARR), University of Sheffield, Regent Court, S1 4DA, Sheffield, UK; Sheffield Centre for Health And Related Research (ScHARR), University of Sheffield, Regent Court, S1 4DA, Sheffield, UK

**Keywords:** inequalities, community health promotion, empowerment, qualitative methods, capacity building, asset-based approaches

## Abstract

Initiatives to promote health and reduce inequalities in place-based communities have increasingly adopted asset-based approaches (ABAs). However, the processes through which such initiatives might reduce inequalities are not well understood, and evidence of their impact on health is still limited. This study aimed to understand how ABAs can impact practices, relationships and the redistribution of resources to reduce health inequalities in and between less advantaged neighbourhoods. Qualitative research was conducted in two settings (England and Spain) where similar asset-based initiatives, aimed at training community members to become health promoters, were being implemented. Data were collected using theory of change workshops, 120 hours of observations and semi-structured interviews with 44 stakeholders (trained community members, voluntary and community sector organizations’ workers and health professionals). A thematic analysis informed by systems thinking was carried out. Three main processes of change were identified: first, ‘enabling asset-based thinking’ defined as supporting people to adopt a view that values their own resources and people’s skills and expertise. Second, ‘developing asset-based capacities’, described as developing personal skills, knowledge, self-confidence and relationships underpinned by asset-based thinking. Finally, ‘changing decision-making and wider health determinants through ABAs’ referred to achieving changes in neighbourhoods through mobilizing the asset-based capacities developed. These processes were associated with changes at an individual level, with potential to contribute to reducing inequalities through supporting individual empowerment and social capital. However, contextual factors were found key to enable or hinder changes in the neighbourhoods and acted as barriers to processes of collective empowerment, thus limiting ABAs’ impact on health inequalities.

**Contribution to Health Promotion**

- A key process in ABA centres on encouraging people to adopt asset-based thinking: valuing themselves and the skills and capabilities of other people.
- This asset-based thinking underpins the development of personal capacities (skills, knowledge, self-confidence and respectful relationships), which community members can use to engage in activities and to support other community members.
- Change processes associated with ABAs enhance individual empowerment and social capital, but in a non-supportive institutional context, can be disempowering.
- When people involved in ABAs have dedicated time to work on community projects and have institutional support and funding, they can achieve wider changes in neighbourhoods sustained over time.

## BACKGROUND

In recent years, initiatives to promote health in high-income countries have increasingly adopted asset-based approaches (ABAs) to address health inequalities in and between place-based communities (Martin-Kerry *et al*., 2023). ABAs centre on supporting people to identify and mobilize resources and relationships (assets) available within places, which can support their health and well-being (Rippon and South, 2017), and ‘make the best use of these resources’ (Cassetti *et al*., 2020, p. 15). Asset-based initiatives take up a variety of forms (Cassetti *et al*., 2020), depending on the contexts and assets available. This asset-based view of people and resources reflects one of the main theoretical paradigms underpinning ABAs, Antonovsky’s salutogenesis (1996), which encouraged a shift of focus from risk factors to looking at protective factors for health. There are still no specific guidelines on how to implement ABAs for public health, but there has been significant theoretical contribution regarding principles and practices (Morgan and Ziglio, 2007; Foot, 2012; Hopkins and Rippon, 2015), namely the importance of mapping available assets and mobilizing these to co-produce a context-specific health intervention (Martin-Kerry *et al*., 2023). According to a recent review of ABAs’ implementation (Cassetti *et al*., 2020), there are three ways to mobilize assets: (i) connecting assets already available to create new activities or partnerships, (ii) raising awareness of available assets that may be underused and/or unknown to the community and (iii) enabling assets to thrive, through supporting people and communities to gain more capacities to increase control over their health determinants. These approaches have been used separately or in combination and underpin ABAs’ implementation in place-based communities (Cassetti *et al*., 2020).

Despite their increasing popularity as an approach to promote health and reduce inequalities (Rippon and South, 2017), evidence that effectively identifies changes attributed to ABAs and their impact on health inequalities is still limited (Van Bortel *et al*., 2019; de Andrade and Angelova, 2020; Peters *et al*., 2021). This might be due to relatively ‘recent’ introduction of ABAs in public health and due to the different paradigms that underpin ABAs, ranging from Antonovsky’s salutogenesis, to the Ottawa Charter for health promotion (WHO, 1986) and community development approaches (Labonte, 1999). Nonetheless, existing literature suggests that changes associated with asset-based initiatives can be understood as ongoing processes of empowerment leading towards improving a person’s and/or population health (Cassetti *et al*., 2020). While it is important to note that the concept of empowerment is complex, many definitions centre on people, individually or collectively, building skills confidence and knowledge to influence decision-making about health and its determinants (Whitehead *et al*., 2016); hence, there is a clear intersection with the underpinning principles of ABAs of supporting people to gain capacities to act in health-promoting ways (Martin-Kerry *et al*., 2023).

However, the distinction between individual and collective empowerment should be taken into account. Wallerstein differentiated changes associated with empowerment into psychological, organizational and community empowerment [(Wallerstein, 1992), p. 198]. Psychological empowerment impacts at individual level and is related to issues such as ‘self-efficacy to act’, ‘belief in group action’ and trust. Organizational empowerment refers to promoting well-functioning services and effective partnership work although it is still linked to an individual dimension, as it refers to empowered workers aiming to re-orient their organization to achieve changes at collective level. Finally, community empowerment includes more collective dimensions such as ‘increasing local action’, ‘stronger social networks’ or equitable access to services [(Wallerstein, 1992), p. 201]. When researching ABAs’ impact on place-based inequalities, it is therefore important to explore how forms of ‘individual’ empowerment can relate to impacts at ‘collective’ levels. Significantly, to effectively address health inequalities, change in empowerment is needed at both individual and collective levels, since power is unequally distributed within societies, as discussed by Popay *et al*. (2021). The study by Popay *et al.* (2021) showed how empowerment can be operationalized by identifying ‘empowerment’ as the process of developing capacities and ‘control’ as the process of using these capacities to effect change. The authors also developed a framework of empowerment, differentiating power in terms of capacities to exercise such control: power within communities (*power within*) across communities (*power with*); *power to* achieve changes; and *power over*, enacted upon communities (Popay *et al*., 2021). The authors finally discussed power in its negative forms, naming this ‘*limiting power*’. This refers to four forms of power, embedded in societies, which act against individual and collective capacity to exercise control, for instance through policies, organizational norms, systematic differences in social hierarchies and marginalization of certain practices by creating stigma through social discourses (Popay *et al*., 2021). In summary, processes of empowerment, which can affect health inequalities in place-based communities, remain difficult to capture, given their multiple characteristics in practice. Combining the implementation of asset-based initiatives to the process of empowerment therefore needs a more in-depth understanding of which forms of empowerment ABAs can promote and how these can be effectively linked to changes at community level, which could ultimately reduce health inequalities within and between places.

A final point should be highlighted, in relation to studying asset-based initiatives’ impact when implemented in communities. Asset-based initiatives build upon local assets, making local contexts central (Orton *et al*., 2017), as what is valued as promoting health in one context may not be the same in another (Foot, 2012). Asset-based initiatives in place-based communities are therefore an example of ‘complex’ interventions, for being multi-component and for their interaction with the wider context where they are being implemented (Kavanagh *et al*., 2022). Such complexity makes it challenging to evaluate the impact that ABAs can generate (South, 2014; de Andrade and Angelova, 2020) and calls for methods that can capture such complexity. To respond to these challenges, systems thinking approaches and qualitative methods have increasingly been adopted to study complex interventions, implemented in complex settings such as communities (Hawe, 2015; Breuer *et al*., 2016; South *et al*., 2020). Systems thinking approaches aim to look at initiatives as taking place within systems as a whole, rather than distinguishing between isolated elements (Hawe *et al*., 2009). In other words, initiatives can be thought of as events and activities that change the system because they interact with it, and new capabilities are created from this interaction, leading to changes in the relationships between actors within the system (Hawe *et al*., 2009). This view of initiatives and change was considered helpful to understand the complexity of asset-based initiatives, given that activities, asset mobilization and potential ‘outcomes’ tend not to be discrete and identifiable but are rather part of a continuous change process (Cassetti *et al*., 2020; Martin-Kerry *et al*., 2023). Hence, adopting a system thinking approach and using qualitative methods, this study aimed to understand how ABAs can impact practices, relationships and the redistribution of resources to reduce health inequalities when implemented in less advantaged neighbourhoods.

## METHODS

### Site selection

Two initiatives were selected as exemplar cases of asset-based initiatives implemented in two countries (Valencia, Spain and Sheffield, UK) where ABAs have been endorsed as part of national policies for health promotion (MSSSI, 2013; PHE, 2015). Both initiatives aimed to train people living or working in less advantaged neighbourhoods to become health promoters, through working in partnerships with local voluntary and community sector organizations (VCS), which identified local volunteers to be trained during a 4- to 6-month training course (Willis and Mustaphanin, 2013; Generalitat Valenciana, 2016) (see Supplementary File 1 for further details on the two initiatives). Following systems thinking approaches, local neighbourhoods were understood as being part of a wider city with its socio-economic and political structures — the ‘system’ — which influence both how services are designed and delivered locally and how professionals such as primary health care (PHC) providers and VCS organizations can work in local neighbourhoods.

In Spain, the initiative also works in collaboration with PHC professionals to plan health promotion activities in the neighbourhoods where it is implemented. This is additionally supported by employing lay health workers (LHWs), lay people who work in neighbourhoods, sharing health information with community members and supporting VCS organizations to organize health promotion activities. The international comparative allowed to explore whether initiatives with similar components work through similar processes and can or cannot lead to similar results even when implemented in different contexts.

### Ontological position and reflexivity

The researcher’s ontological position reflects subtle realism: ‘an external reality exists, but is only known through […] socially constructed meanings’ (Ormston *et al*., 2014, p. 5). To explore these social realities, being a foreigner in both settings, and not representing the programmes helped developing a collaborative relationship with most of the other participants and was perceived to, at least partially, circumvent existing differences in socio-economic background when working with less advantaged participants.

### Data collection and analysis

Data collection and analysis were organized in three main stages. First, an initial theory of change (TOC) was developed to explore the implicit and explicit processes of change underpinning the selected initiatives and anticipated changes in each local system (Breuer *et al*., 2016) and to understand the complexity of both the programmes and the ‘systems’ where these are embedded. In each context, a purposive sample of stakeholders, who had been involved in the design or implementation of the initiative, was identified through discussion with the local coordinators and invited to a TOC workshop. The TOC workshops were transcribed and analysed to identify activities to observe and stakeholders to interview. They also allowed the researcher to explore the expected impact of both programmes and thus include questions related to these in the subsequent interviews. This data informed the second main stage of data collection and analysis: 5-month fieldwork in each setting, which took place between March 2018 and April 2019. Participants were recruited through initial contact via the programme managers, followed by a snow-balling approach. A total of 44 semi-structured interviews and 120 hours of participant observations were carried out in the two settings, to explore how the initiatives were ‘embedding’ in each place (Hawe *et al*., 2009), and the change processes resulting from the implementation of the asset-based initiatives, as perceived by involved stakeholders, being these current and former learners in the training for lay people included in both programmes, representatives of VCS organizations, PHC professionals or local council members collaborating with the two initiatives (see Supplementary File 2 for details on data collection). The identified changes were also compared to the expected impacts described in the TOC (available as Supplementary Files 3 and 4). This research was part of a doctoral programme, and a literature review was previously published (Cassetti *et al*., 2020). The research was approved by the Ethics Committee of the School of Health And Related Research of the University of Sheffield (now Sheffield Centre for Health and Related Research).

Third, data from interviews were transcribed verbatim and, together with the fieldnotes, analysed with NVivo Software v12 using thematic analysis (Braun and Clarke, 2012) informed by Hawe’s framework (2015) about evaluating community-based initiatives that suggest to explore: (i) how the initiative interacted with local context and in what ways decision-making processes and/or practices may have changed; (ii) how relationships may have changed; (iii) changes in activities; and (iv) changes in the redistribution of resources. Hawe’s framework informed the organization of codes into themes and helped organize the processes of change identified. The analysis was carried out within and across cases, by comparing emerging codes within and across the settings, to identify processes of change associated with each asset-based initiative, to explore potential cross-case patterns, and how asset-based initiatives and context were interacting and co-evolving (Orton *et al*., 2017). Interestingly, despite the different settings, the analysis identified mostly similar change processes in both settings, although contextual factors acted as barriers and/or facilitators in different ways, as will now be discussed.

## RESULTS

The analysis identified three main processes of change that explained how ABAs were working in both contexts. First, ‘enabling asset-based thinking’ defined as supporting people to value their own skills and resources and other resources available in their community, including people’s skills and expertise. Second, ‘developing asset-based capacities’ understood as a process of developing personal skills, knowledge, self-confidence and relationships underpinned by asset-based thinking. Finally, ‘changing decision-making and wider health determinants through asset-based approaches’ refers to a process of achieving wider changes by mobilizing the developed individual asset-based capacities beyond the time and space of the initiatives. However, following systems thinking approaches, local contexts (the ‘systems’) were central in the analysis, which also identified how contextual factors could act as barriers or enablers for changes to occur, an important finding when it comes to understand the potential impact of ABAs on place-based inequalities.

 Please note that, in reporting direct quotes, the following labels will be used to identify the different stakeholders: lay health volunteers (LHV) referring to stakeholders who are being trained or have been trained; voluntary and community sector workers (VCS) referring to former learners currently working in local organizations, and health professionals (HP).

### Enabling asset-based thinking

The first process, ‘enabling asset-based thinking’, describes how people were supported to value themselves, the people around them and their communities, rather than focusing on problems or deficits. The activities within the courses were designed to support learners to develop asset-based thinking, which emerged to different degrees and at different times. First, both courses included an asset-mapping activity, for participants to reflect on their neighbourhoods. This allowed learners to initially discuss assets in terms of tangible resources, such as community organizations or places (libraries, gyms). Following this, by creating space for peer- and tutor-learner debates, learners were encouraged to value each other’s skills and knowledge, resulting in learners starting to feel valued and expressing more positive views about themselves and others:

LHV8—Valencia: The course was like a therapy, you know? When something hurts, you have a scar and you put on cream and it heals. For me it was a therapy, to achieve this. It means a lot to me.

Researcher: what do you feel that it has given you?

LHV8—Valencia:… “you can!” The messages [received] in the training. That really got to me…[…]. “You can”, this message was a very strong message for me.

However, adopting asset-based thinking was not without difficulties. In both contexts, neighbourhood stigma (‘negative’ labels to refer to the communities where the initiatives took place) appeared to shape this process. Learners and professionals working locally claimed that labelling areas or people using negative labels can generate negative feelings and perspectives in local people, as well as among professionals working in that area, making it harder for people directly engaged with the initiatives to adopt the asset-based thinking:

The concept of [neighbourhood X] is that of a very conflicting neighbourhood, very marginalised. When I said that I had been assigned here, people put their hands on their heads. But I tell you the truth, I love it. I’m very happy. (HP7-Valencia)

You know, people don’t want to be labelled with ‘deprived area’ because that’s all they have been labelled with. It might be deprived of funds, but we are very rich in culture, we are very rich in experience, in everything else we do. Why are we just labelled as areas of high deprivation? (VCS4-Sheffield, former learner)

Working against this pre-existing stigma, adopting an asset-based thinking, through valuing people, skills and resources, sets the basis for processes of capacity development, as included in the second theme.

### Developing asset-based capacities

The second process — ‘developing asset-based capacities’ — involved: (i) developing new personal skills and knowledge, (ii) increasing self-confidence and (iii) developing new relationships, and was underpinned by the adoption of asset-based thinking. Learners in both settings began to strengthen or develop new skills and knowledge during the training. This included, for example, increasing their knowledge about what can influence health, or services available where they live and how to access these. Learners also started to develop skills for group working or speaking in public:

The course did help me, because you learn how to run workshops, standing up in front of people, I had never done that, quite the contrary. (LHV1-Valencia)

Increasing skills and knowledge reflected the objectives of the training courses, as discussed in both TOC workshops. However, these changes, in turn, supported learners in gaining self-confidence, as most learners started to feel capable of sharing their views with others, feeling less shy and believing more in themselves:

I was married very young and probably don’t expect I was able to succeed at education and ICDH, along with other factors, was part of what made me think “I can do this” [a university degree]. It gave me the confidence. (VCS4-Sheffield)

Finally, developing asset-based capacities involved developing relationships, where all people were valued for their skills and expertise. The courses created spaces to meet new people from a variety of sociocultural backgrounds, which not only allowed for new relationships to be developed across those pre-existing boundaries but also allowed for relationships where the other person was valued for her/his expertise and cultural background:

I just always remember back [when I was] a participant [in ICDH], and what I really liked about the course was that real mixed [of] people […] I think that was the strength of the course. (VCS3-Sheffield)

In Sheffield, asset-based relationships were also developed between people in the course and professionals working in the local neighbourhoods. Former learners reported that towards the end of the course, a session was dedicated to meet representatives of local VCS organizations:

Researcher: after the ICDH you got the job at the [VCS organisation]?

LHV2-Sheffield: yes, the guy from [VCS organisation] came to the ICDH, to tell us about the community activities they were doing, that’s how I met him.

In Valencia, through the community work carried out within the initiative, additional asset-based relationships were developed outside of the courses between LHWs, health professionals and VCS organizations. For instance, health professionals established new asset-based relationships with VCS organizations working in their area:

With the local associations, we have a relation, which we did not have before. (HP2-Valencia)

although dedicating time to develop those relations was key:

It’s about going, and going and going, so they get to know you. (HP3-Valencia)

In summary, asset-based capacities became an example of a process of empowerment, aiming to challenge pre-existing unequal relationships across different stakeholders in communities, allowing people to believe more in themselves and act on community networks as a determinant of health. Developing asset-based capacities sets the basis for further changes, potentially impacting the wider community, as the last theme shows.

### Changing decision-making and wider health determinants through asset-based approaches

The third process, ‘changing decision-making and wider health determinants through asset-based approaches’, referred to how learners (and health professionals in the case of Spain) were able to mobilize the acquired asset-based capacities within their communities or workplaces, taking collective action to influence decision-making and wider determinants of health. For instance, there were examples of former learners developing new activities and local events to foster social relationships:

We worked alongside [VCS organisation] and the church, we did a community street event. […] like “bring a dish”, so everybody who came, brought a dish, brought their own culture food and you know…that’s how it happened… (VCS1-Sheffield)

There were also examples of learners supporting other local community members in becoming aware of available services, through sharing information acquired in the course among marginalized social groups, thereby addressing processes of exclusion within the existing health system:

In this Gypsy Romanian community, there’s a lot of lack of information. A lot of things happen because there is a lack of information […] Now I have this information here, with me. And this is what I share [with others]. (LHV8-Valencia)

Another important change related to mobilizing the developed asset-based relationships between organizations where former learners and health professionals (only in Spain) were working. This enhanced opportunities for partnership work and resulted in collaborations to develop new activities in the community where former learners and professionals work, addressing deficits in health promotion within the existing health system. For instance, organizing cancer support in a neighbourhood in collaboration with another VCS organization:

We’ve been working with [VCS organisation], who treat people with cancer. And we’ve got a disproportionate number of people who get cancer in our area. […] We can’t deliver that, what they deliver. So, we’ve been working with them, to put a bid in, to come and do some outreach in our area. (VCS2-Sheffield)

Similarly, through the community work as part of the Spanish initiative, health professionals reported developing *ad hoc* collaborations with local VCS organizations to deliver health education workshops, tailored to the needs expressed by the VCS organizations:

At a meeting between nurses, MIHsalud staff and the organisation, aimed at presenting the possible health workshops which the health centre can offer:[…] the organisation was interested in workshops related to sexually-transmitted infections and gender equality, as last year those [workshops] went very well. [observation notes-Valencia]

Again, contextual factors played an important role in favouring or hindering how different people engaged in the initiatives in both settings could deploy their personal capacities to achieve changes with potential for collective impact. The lack of dedicated time and funding for partnership work countered the potential for a greater impact in workplaces and practices. For example, because funding opportunities tend to encourage organizations to compete against each other:

The MIHsalud programme does facilitate that union [between organisations], but…we sit all together, exchange experience, but then everyone has their own “shopping”. (LHV10-Valencia)

As for changes in workplaces, few former learners reported being able to use the developed skills in their job:

The learning and teaching methods [of ICDH] were fantastic skills I learnt. [I’m now] using different methods to [support my users]. (LHV1-Sheffield)

Nonetheless, not everybody was able to work in jobs that required those skills and knowledge:

It is another type of work, we do individual interviews and individual follow-up. We have done the workshops during the course […] but not anymore. (LHV5-Valencia)

An important change in workplaces was, however, identified when learners were able to embed the asset-based thinking to their work practices and culture, a change at personal level (change in attitudes) that, at the same time, could impact at collective level because of the ways work was reorganized or delivered. For instance, a former learner currently working in a VCS organization in Sheffield commented that the ICDH contributed to changing attitudes within the organization:

[the ICDH course] changed my way of thinking and doing, you know, [from] ‘this user can’t do it’ to ‘actually yes they can do it, who says they can’t.’ [...] the ICDH course… it kind of just [makes] people think differently, when they are planning. (LHV6-Sheffield)

As for health professionals engaged in the community work in Valencia, transferring the asset-based thinking into their workplace resulted, for some of them, in changing their attitudes towards the communities they work in, resulting in them engaging more:

In fact, thanks to the MIHsalud intervention, we are now paying attention to the community. Because the health professionals are used to people coming to the health centre, and in primary care is not like that. I am the one who has to reach out. Through the MIHsalud programme, we have managed to go out in the community [refers to dedicating two working afternoon of two nurses to go out to talk with associations and local shop owners]. (HP6)

It is challenging to attribute the changes presented here ‘only’ to the implementation of the asset-based initiative, yet in interviews, former learners discussed these examples as being related to the asset-based capacities they acquired in their training; although not explicitly mentioning ‘asset-based capacities’ but, for example, through discussing increased self-confidence, acquired knowledge or awareness, or new relationships made through the courses:

[The ICDH] has built up my confidence not to work on one-to-one but to work in the wider community, a wider range of culture. (LHV1-Sheffield)

Being able to mobilize asset-based capacities thus became a form of increasing control over some of the wider social determinants of health, specifically relationships and the organization of services, even though most of the changes described depended on their interaction with local factors that could act as barriers and become disempowering. For instance, the importance of receiving institutional support was central in the possibility for former learners, and health professionals directly engaged with the initiatives to mobilise the acquired asset-based capacities within their workplace. Despite formal support for the initiatives, both health professionals in Valencia and former learners working in VCS organizations in Sheffield discussed lacking recognition for their work in communities. In both cases, participants felt that their colleagues or managers perceived community work as similar to going out shopping, making it difficult for them to dedicate time to develop relationships with communities and providing ‘evidence’ of the impact of their work among colleagues:

HP1 Valencia: ‘And then, [colleagues] started talking.

HP2: yes, ‘they are going out in the streets’ ‘they are there all day’.

HP1: as if we were shopping. So we did a session to explain what we were doing

Our hands are tied so much… if I send someone around, they’ll think we are gonna go and shop, doing our own shopping. There is so much mis-trust. Coz they don’t understand it. (VCS7-Sheffield)

The importance of formal support had also been discussed by staff during the TOC workshops in both settings, who argued that ensuring ‘institutional support’ by their manager [TOC workshop Spain] and the ‘political will’ of local government [TOC workshop UK] were fundamental for the initiatives to exist and for their continuity.

**Fig. 1:**
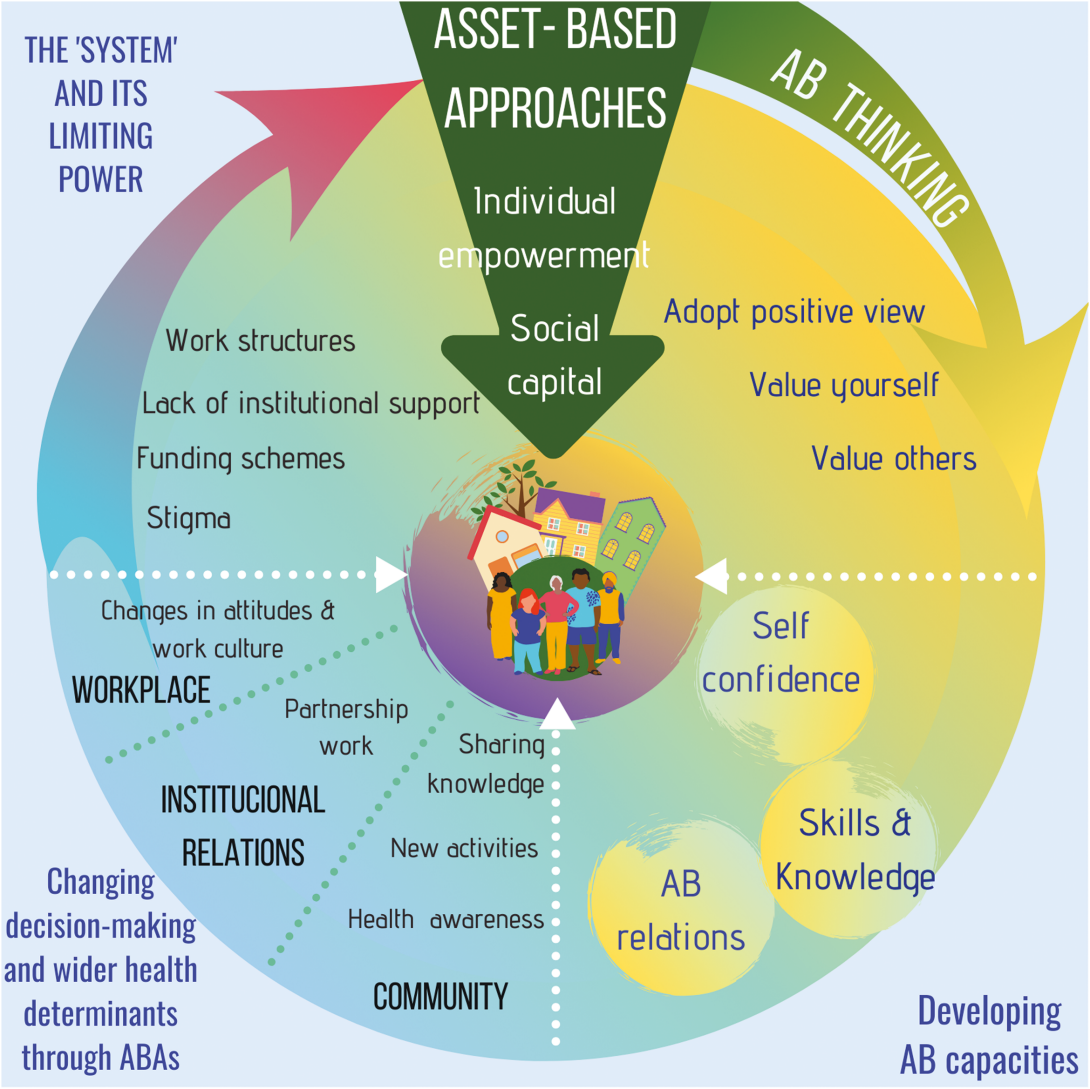
Processes of change associated with implementing asset-based approaches in communities (authors' own).

In conclusion, even though both asset-based initiatives provided learners and health professionals with new capacities to act on some of the wider health determinants through mobilizing asset-based capacities, contextual factors were key to understanding the changes that ABAs can generate. Figure 1 visually presents the process of change, which ABAs can enable in communities, based on the analysis and interpretation of the data from this study. These are important findings when it comes to unpacking the potential of ABAs to reduce inequalities.

## DISCUSSION

This research analysed two examples of asset-based initiatives, in England and Spain, to understand how ABAs can impact practices, relationships and redistribution of resources to support reducing health inequalities experienced in less advantaged neighbourhoods. It identified three main processes of change: ‘enabling asset-based thinking’ defined as supporting people to adopt a view that values their own resources and people’s skills and expertise; ‘developing asset-based capacities’ described as developing personal skills, knowledge, self-confidence and relationships underpinned by asset-based thinking; and ‘changing decision-making and wider health determinants through ABAs’ referred to achieving changes in neighbourhoods through mobilizing the developed AB capacities.

This research found that the adoption of an assets’ perspective, defined here as asset-based thinking, was a core process of ABAs, common to both settings. This reflects previous work on ABAs, which discusses the need for a change of mindset: from a deficit-based view to an asset-based view (Foot, 2012; Hopkins and Rippon, 2015). However, the findings from this study suggest that this change of mindset is a core practice and process through which ABAs can promote health in place-based communities, which underpins the other change processes: namely, the development and mobilization of asset-based capacities to enable changes in decision-making and the wider health determinants in local communities.

Importantly, to respond to the aim of this study regarding understanding how ABAs can impact practices, relationships and redistribution of resources, this study found that most changes associated with the implementation of ABAs related to developing personal capacities in the form of skills, knowledge and relationship-building. Previous research has shown that developing capacities is a process, which can promote health through supporting people to acquire knowledge and skills to tackle health determinants and increase control over decisions affecting their health (Liberato *et al*., 2011), which can be understood as a form of empowerment (Wallerstein, 1992). In this study, learners increased their knowledge about existing health services (Tengland, 2006), and how to better access those services (Whitehead, 2007). Hence, both forms of knowledge can be seen as a step towards individual empowerment, as evidence suggests (Goodman *et al*., 1998). Additionally, when mobilizing the acquired asset-based capacities, some of the learners took up new roles in their communities, for instance when developing local activities or when sharing the acquired knowledge among their networks, becoming a sort of ‘role-model’ whom other community members could approach to seek support while organizing health-promoting events or could ask for help on health-related issues. Taking up new roles is a way to change one’s own self-image, which also becomes a form of individual empowerment (Tengland, 2006; Allen *et al*., 2022). At the same time, engaging in local activities or sharing health information (Ure *et al*., 2021) can potentially contribute to increase ‘power within’ a group (Popay *et al*., 2021, p. 1258), in the form of collective knowledge about how to tackle health determinants.

Another important change associated with ABAs was that the courses provided a space for people from different backgrounds to develop relationships that ‘bridged’ differences between them and to value each other, share knowledge and support each other when in need (Poortinga, 2012; Adams, 2020). This can be seen as an example of developing bridging social capital, which can improve and protect health through supporting people, so that they feel cared for (Villalonga-Olives and Kawachi, 2017) and, in this case, with the addition of feeling valued. It also supports increasing the network diversity within communities, which can contribute to promote power to act ‘with’ others to address the wider health determinants, beyond social differences (Popay *et al*., 2021, p. 1258). These changes are therefore examples of ways through which ABAs can promote people’s empowerment and social capital.

However, an important contribution of this research is that adopting a systems thinking perspective helped to demonstrate how contextual factors (neighbourhood stigma, lack of funding for intersectoral work, lack of institutional support and general work structures) enabled or hindered the mobilization of asset-based capacities beyond the time and space of the initiatives, for the different people engaged in the initiatives, and the extent to which the identified changes could affect health inequalities experienced and the wider health determinants in both neighbourhoods. An important barrier relates to the ways in which ‘limiting power’ shapes contexts, limiting the impacts that ABAs aim to generate; for instance, via the way stigmatizing and negative labels used for neighbourhoods (a form of ‘productive power’) can disempower and disadvantage (Popay *et al*., 2021, p. 1258), negatively impacting self-perceived health among people who live there (Halliday *et al*., 2020). Likewise, although developing new activities in the community or sharing health information about health, and healthcare access and use, with other community members may support some community members, inequalities in access to services may not necessarily be reduced, as these are determined by other limiting powers, such as the systems’ social and political structures. Finally, these ‘limiting powers’ also acted at institutional level, becoming a form of what has been defined as ‘institutional power’: a more subtle form of power operating through existing organizational system activities [(Popay *et al*., 2021), p. 1258]. For instance, current funding schemes favour competition among organizations, and traditional single-sector work cultures make partnership work challenging, as reflected both in this research and in other recent studies (Boydell and Rugkåsa, 2007; Kavanagh *et al*., 2022). Similarly, trying to re-orient work practices towards an assets’ perspective was found to depend on being institutionally supported by managers: health professionals in Valencia were able to develop new activities with local VCS organizations only when supported by their coordinator. This reflects a key point made by Hopkins and Rippon (2015) who highlighted that, for assets to be mobilized, there needs to be supported by system leaders who can create the conditions for a shift towards ABAs, thus changing the current organizational structures. All these examples of limiting power can counter the potential of ABAs in reducing health inequalities as they limit the extent to which the identified change processes can effectively impact wider health determinants, which are generally most responsible of reproducing systematic health inequalities in and between place-based communities.

In conclusion, as these findings show, and contrary to what other authors have suggested (Zimmerman, 2000), individual empowerment was developed through the ABAs, but this alone had limited power over changing the wider social determinants within neighbourhoods in Sheffield and Valencia. These findings reflect those of Egan *et al*. (2021) who studied how community empowerment initiatives could support collective agency and health and found that systems’ structures, such as unequal access to resources or state disinvestment in local areas, acted as barriers to achieve changes that could affect health inequalities. Similarly, Nickel and von dem Knesebeck (2020), in their review of community-based health promotion and prevention programmes, found that community-wide initiatives showed limited evidence of changes at collective level and that contextual factors might affect such limitation. Hence, the importance to account for the wider contexts where initiatives are being implemented when researching how these can affect health inequalities in place-based communities.

A final point should be made as to the value of the methodological approach chosen for this study. Taking a systems perspective allowed the researcher to contrast the identified changes with contextual factors, which have often been neglected when evaluating public health interventions (Orton *et al*., 2017; South *et al*., 2020). Through a systems approach, ABAs can be conceptualized as ongoing processes, with the findings showing that changes identified by participants, even when initiated in the past, still influenced their views and current practices, thus generating other (unanticipated) ongoing changes. However, it also helped to see the ways in which people were disempowered in their communities because of contextual factors (e.g. due to stigma, lack of job opportunities, limited support in workplaces and limited fundings for partnership work), which countered the potential impact of ABAs.

To conclude, taking into account the systems where these initiatives are implemented showed that even though ABAs can support people to increase control over their health and some of its determinants, without more sustained action on the wider social determinants of health, the impact of ABAs remains limited or might even be countered. This is in line with previous critiques of ABAs, namely its lack of accounting for the unjust causes of inequalities where ABAs were proposed (Friedli, 2013).

### Strengths and limitations

An important strength of this study is that it is the first to compare two asset-based initiatives implemented in contrasting settings, which has proven to enhance the understanding of the processes through which ABAs work by considering its different implementations. Nonetheless, although this study has tried to be as comprehensive as possible, there are some limitations worth mentioning. First, to have a wider perspective on the impact of the initiatives in the neighbourhoods where they are implemented, the sampling could have benefited from including people not directly engaged with the initiative, which was not possible for reasons of time and difficulties in accessing those potential participants. Second, the limited sample affects the transferability of the findings. However, it still provides insight which are potentially transferable to other AB initiatives in contexts sharing similar characteristics. For instance, the findings related to the processes identified in this research (developing and mobilizing AB capacities to enable change), as key processes of change, may be transferable, as these may be processes that arise from other AB initiatives, even though the capacities developed might be different to those identified in this study.

## CONCLUSION

This study aimed to understand how ABAs can impact practices, relationships and the redistribution of resources to reduce health inequalities experienced in less advantaged neighbourhoods through qualitative research into two asset-based initiatives implemented in two different contexts (Valencia and Sheffield). A core process in ABAs was identified, ‘enabling asset-based thinking’, which underpinned other changes in people and communities derived from developing and mobilizing asset-based capacities. It showed that these changes can empower people and increase social capital. However, it also found that contextual factors can hinder changes at a more collective level, thus limiting the potential of ABAs to affect health inequalities. These findings suggest that future planning of ABAs should ensure that the asset-based thinking needs to be adopted, although the forms through which this can occur depend on the contexts and stakeholders involved. Likewise, it is important to ensure that system leaders support the asset-based initiative: without this, its impact risks being limited to the goodwill of interested individual and sustainability undermined.

Additionally, this study contributes to the understanding of ABAs from a methodological perspective, highlighting how systems thinking approaches can be helpful to analyse the complexity of these initiatives. Further research could usefully focus on identifying practices that take into account contextual factors in ways that enhance the potential of ABAs and the achievement of changes at a more systems level, thus impacting on wider health determinants.

## Supplementary Material

daae017_suppl_Supplementary_Files_1

daae017_suppl_Supplementary_Files_2

daae017_suppl_Supplementary_Files_3

daae017_suppl_Supplementary_Files_4
