# Supplementary material for: How can asset-based approaches reduce inequalities? Exploring processes of change in England and Spain
Source: Health Promot Int. 2024 Mar 2;39(2):daae017. doi: 10.1093/heapro/daae017 (PMC10908351; doi:10.1093/heapro/daae017)
Supplement: daae017_suppl_Supplementary_Files_1 [file daae017_suppl_supplementary_files_1.docx]

Supplementary file 1: Description of the aims, target population and activities of the two selected initiatives

| **MIH Salud**  (from its acronym in Spanish: women, children and men promoting health)  Valencia (Spain) | **Introduction to Community Development and Health**  (ICDH, part of the Community Wellbeing initiative)  Sheffield (UK) |
| --- | --- |
| AIM:  To empower people, promote health and reduce inequalities through training local people to become peer health promoters and to foster working in partnerships with local VCS organisations and primary healthcare, involving them in delivering health promotion activities.  TARGET POPULATION  Targeted at VCS organisations and people living in less advantaged areas of Valencia  ACTIVITIES  1.  Five months training on health promotion topics for people volunteering in local VCS organisations to become lay health volunteers (LHVs) and provide peer support in their communities. The training is called a “learning&action” training, as LHVs attend weekly classes and are then required to organise workshops related to the topics learnt. These workshops are carried out in their own VCS organisations, for its members and users.  2.  A once-a-month workshop on a health topic, where VCS organisations come along and learn about each other’s activities, services available or other health-related topics. This is thought to support VCS organisations to become aware of their potential as health-promoting stakeholders  3.  Community work: three community lay health workers (LHWs) are employed to work in the three less advantaged neighbourhoods as community link/connectors, to share health information through informal chats in the streets, distributing leaflets in local shops which are selected for being ‘health info points’ and delivering health workshops.  4.  Monthly meetings with primary healthcare professionals in those three less advantaged neighbourhoods to foster a more community-oriented primary healthcare through raising awareness among health professionals about local assets and how to work to support local people to access healthcare services appropriately | AIM:  To empower people to take control over their own life and to raise awareness on how to promote the health and wellbeing of their communities through adopting community development principles, thus working on developing local assets.  TARGET POPULATION  Targeted at people living in less advantaged areas of Sheffield  ACTIVITIES  1.  A 15 weeks course (the Introduction to Community Development and Health - ICDH) for neighbourhoods’s residents identified by local VCS organisations. The course aims to increase participants’ self-confidence, raise awareness on health and its social determinants, promote empowerment and increase capacities to change and work with others.  2.  Throughout the course, learners become aware of VCS organisations or project in their area where they can volunteer if interested. In some cases, some of the trained people get engaged in their local groups or in the local partnerships at neighbourhood level, where VCS organisations come together to identify needs and actions to support community health |
