# Supplementary material for: How can asset-based approaches reduce inequalities? Exploring processes of change in England and Spain
Source: Health Promot Int. 2024 Mar 2;39(2):daae017. doi: 10.1093/heapro/daae017 (PMC10908351; doi:10.1093/heapro/daae017)
Supplement: daae017_suppl_Supplementary_Files_2 [file daae017_suppl_supplementary_files_2.docx]

Supplementary file 2: Summary of data collection methods and participants

| **Type of participant in interviews** | **No. of participants** | **Location** |
| --- | --- | --- |
| MIHsalud current and former staff members | 5 | Valencia |
| Health professionals | 8 | Valencia |
| Lay Health Volunteers (people in training of former trainees) | 12 | Valencia |
| Current or former Lay Health Workers | 6 | Valencia |
| ICDH tutors and staff member | 5 | Sheffield |
| Former ICDH trainees and currently voluntary and community sector workers | 6 | Sheffield |
| Voluntary and community sector worker | 2 | Sheffield |
| **Type of event observed** | **No. of hours of observations or No. of events observed** | **Location** |
| MIHsalud course | 26 hours | Valencia |
| Workshops delivered by LHV currently being trained | 16.5 hours | Valencia |
| Meetings of MIHsalud team with health professionals | 5 events | Valencia |
| Team meeting | 4 events | Valencia |
| Walking in the neighbourhood with a LHW | 19 hours | Valencia |
| Extra: walking with a previously trained LHV, visiting two local VCS organisations | 3 events | Valencia |
| ICDH course | 36 hours | Sheffield |
| Extra: public events, walking around a neighbourhood, observing an event led by a former learner. | 8.5 hours | Sheffield |
