# Supplementary figures and images for: How can asset-based approaches reduce inequalities? Exploring processes of change in England and Spain
Source: Health Promot Int. 2024 Mar 2;39(2):daae017. doi: 10.1093/heapro/daae017 (PMC10908351; doi:10.1093/heapro/daae017)

Supplementary file 3: initial TOC in England


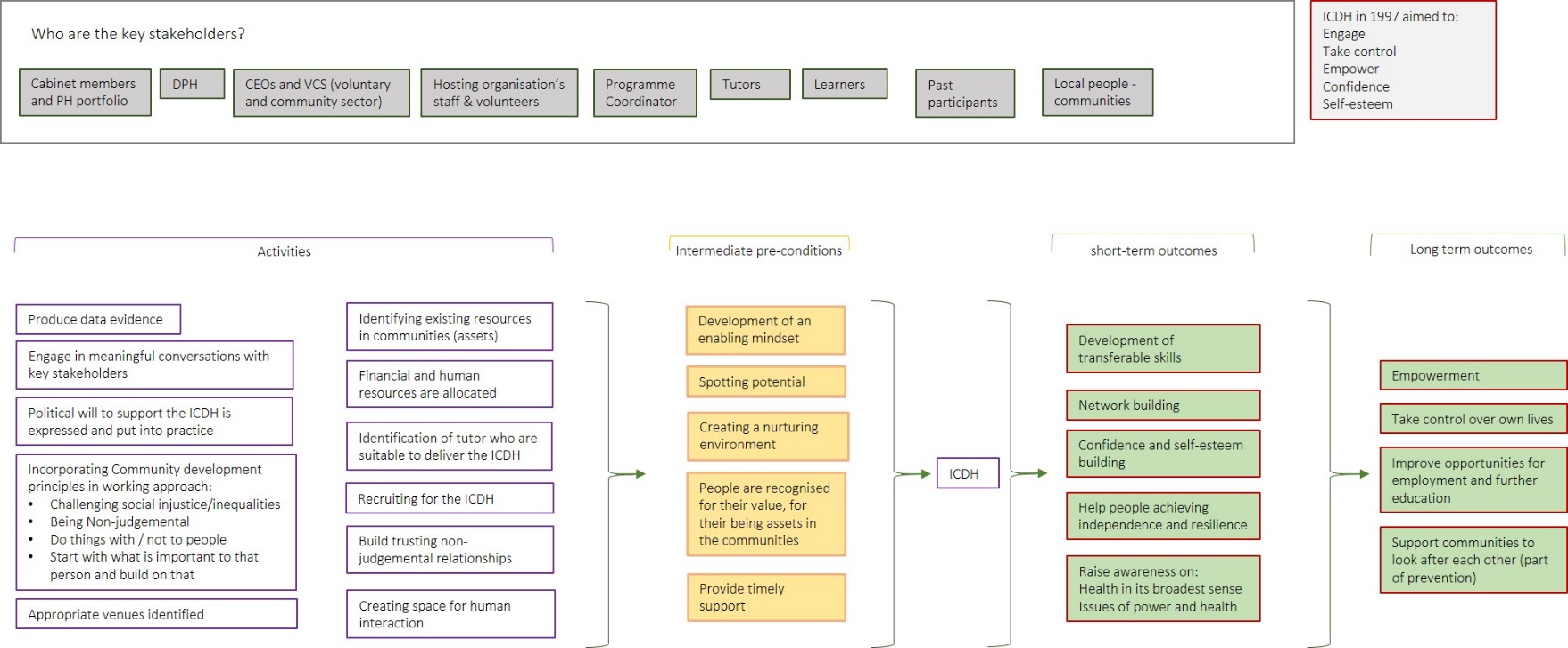

Supplement: daae017_suppl_Supplementary_Files_3 [file daae017_suppl_supplementary_files_3.docx]

Supplementary file 4: initial TOC in Spain


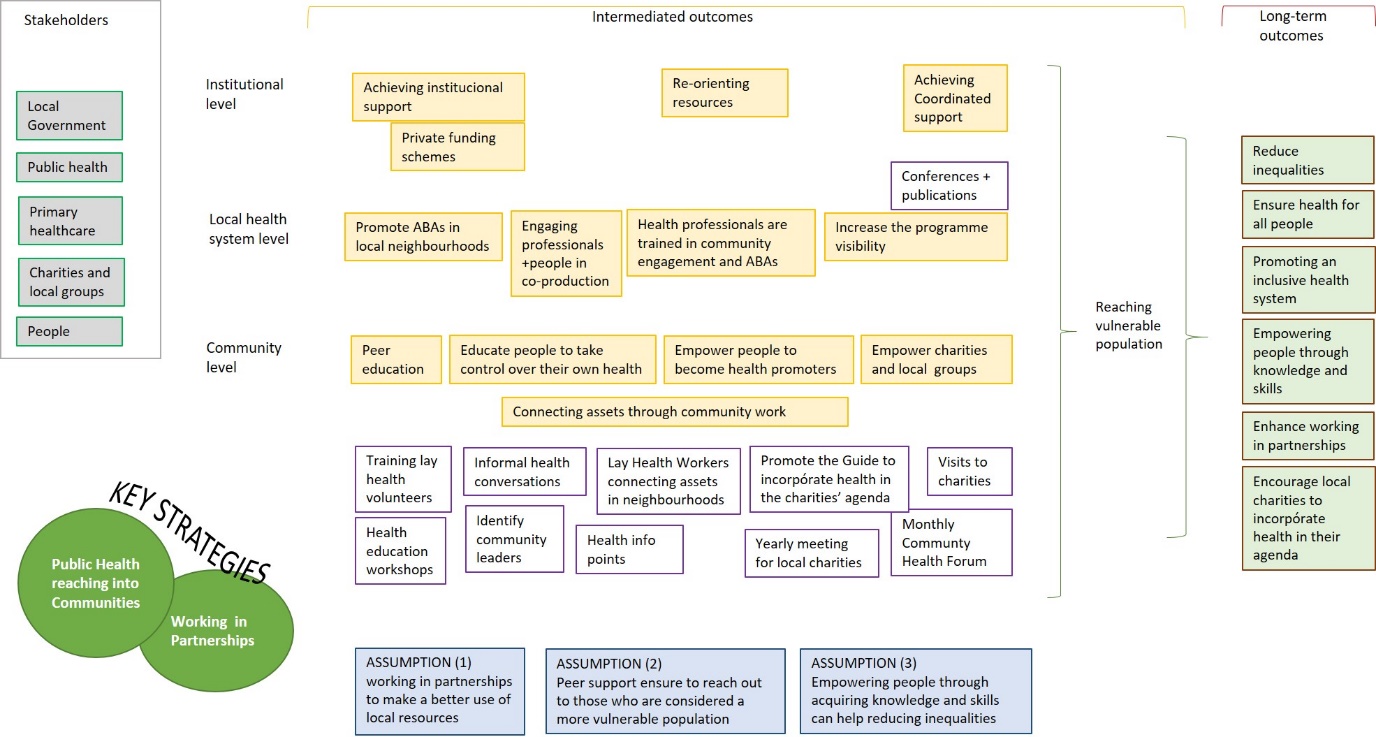

Supplement: daae017_suppl_Supplementary_Files_4 [file daae017_suppl_supplementary_files_4.docx]
